# A glycosylation-related signature predicts survival in pancreatic cancer

**DOI:** 10.18632/aging.205258

**Published:** 2023-11-30

**Authors:** Huidong Hu, Bingsheng He, Mingang He, Hengmin Tao, Baosheng Li

**Affiliations:** 1 Department of Oncology, The Affiliated Hospital of Southwest Medical University, Luzhou 646000, China; 2 Department of Gastrointestinal Surgery, Shandong Tumor Hospital and Institute, Shandong First Medical University and Shandong Academy of Medical Sciences, Jinan 250117, China; 3 Department of Head and Neck Radiotherapy, Shandong Provincial ENT Hospital, Shandong University, Jinan 250117, China; 4 Department of Radiation Oncology, Shandong Cancer Hospital and Institute, Shandong First Medical University and Shandong Academy of Medical Sciences, Jinan 250117, China

**Keywords:** glycosylation, pancreatic cancer, prognosis, risk signature, GRGs

## Abstract

Background: Tumor initiation and progression are closely associated with glycosylation. However, glycosylated molecules have not been the subject of extensive studies as prognostic markers for pancreatic cancer. The objectives of this study were to identify glycosylation-related genes in pancreatic cancer and use them to construct reliable prognostic models.

Materials and Methods: The Cancer Genome Atlas and Gene Expression Omnibus databases were used to assess the differential expression of glycosylation-related genes; four clusters were identified based on consistent clustering analysis. Kaplan–Meier analyses identified three glycosylation-related genes associated with overall survival. LASSO analysis was then performed on The Cancer Genome Atlas and International Cancer Genome Consortium databases to identify glycosylation-related signatures. We identified 12 GRGs differently expressed in pancreatic cancer and selected three genes (SEL1L, TUBA1C, and SDC1) to build a prognostic model. Thereafter, patients were divided into high and low-risk groups. Eventually, we performed Quantitative real-time PCR (qRT-PCR) to validate the signature.

Results: Clinical outcomes were significantly poorer in the high-risk group than in the low-risk group. There were also significant correlations between the high-risk group and several risk factors, including no-smoking history, drinking history, radiotherapy history, and lower tumor grade. Furthermore, the high-risk group had a higher proportion of immune cells. Eventually, three glycosylation-related genes were validated in human PC cell lines.

Conclusion: This study identified the glycosylation-related signature for pancreatic cancer. It is an effective predictor of survival and can guide treatment decisions.

## INTRODUCTION

Pancreatic cancer is one of the most lethal malignancies in the world, with a high death rate and poor prognosis [1]. Annually, there are approximately 57,600 newly diagnosed cases of pancreatic cancer, with 47,050 deaths [2]. Pancreatic cancer survival rates remain low compared with those of other malignancies despite improvements in diagnostic and therapeutic techniques [3]. Therefore, it is increasingly necessary to develop new prognostic indicators that accurately predict the patient’s prognosis and guide treatment.

Protein glycosylation is one of the most important post-translational modifications of proteins. It involves carbohydrate transfer to proteins by glycosyltransferases and glycosidases [4–7]. Glycosylation is generally classified as N-linked or O-linked. N-glycosylation occurs when glycans from lipid-linked oligosaccharides are transferred to asparagine residues in the protein [8], while O-glycosylation occurs when glycans are linked to the hydroxyl groups of serine or threonine residues [9]. N-glycans contain three mannose and two N-acetylglucosamine (GlcNAc) subunits as the common pentasaccharides; this can be modified by adding galactose, GlcNAc, fucose, and sialic acid moieties [10].

Glycosylation aberrations are proposed hallmarks of most cancers [11]. Glycosylation regulates key steps of cancer biology, including tumor invasion, metastasis, and cellular signaling [12–15]. Moreover, abnormal glycosylation is an important indicator to induce tumor immune regulation because it provides recognition antigens for T cells [11, 16]. Abnormal glycosylation patterns identified in pancreatic cancer include O-GlcNAc, sialylation, aberrant branching, O-glycan structures, fucosylation, and altered mucins [17]. This alters multiple tumor-promoting signaling pathways, augments metastatic phenotypes, and remodels the tumor immune microenvironment [18].

Several prognostic models for pancreatic cancer were developed using post-translational regulatory biomarkers, including alternative splicing [19] and N6-methyladenosine modification [20]. Despite being a prominent hallmark of over 300 post-translational modifications [21], no systematic research has been conducted on the relationship between glycosylation and pancreatic cancer. This study developed a glycosylation-based three-gene prognostic model in pancreatic cancer and demonstrated its predictive ability.

## RESULTS

### Identification of differentially expressed glycosylation-related genes in pancreatic cancer

**Figure 1:**
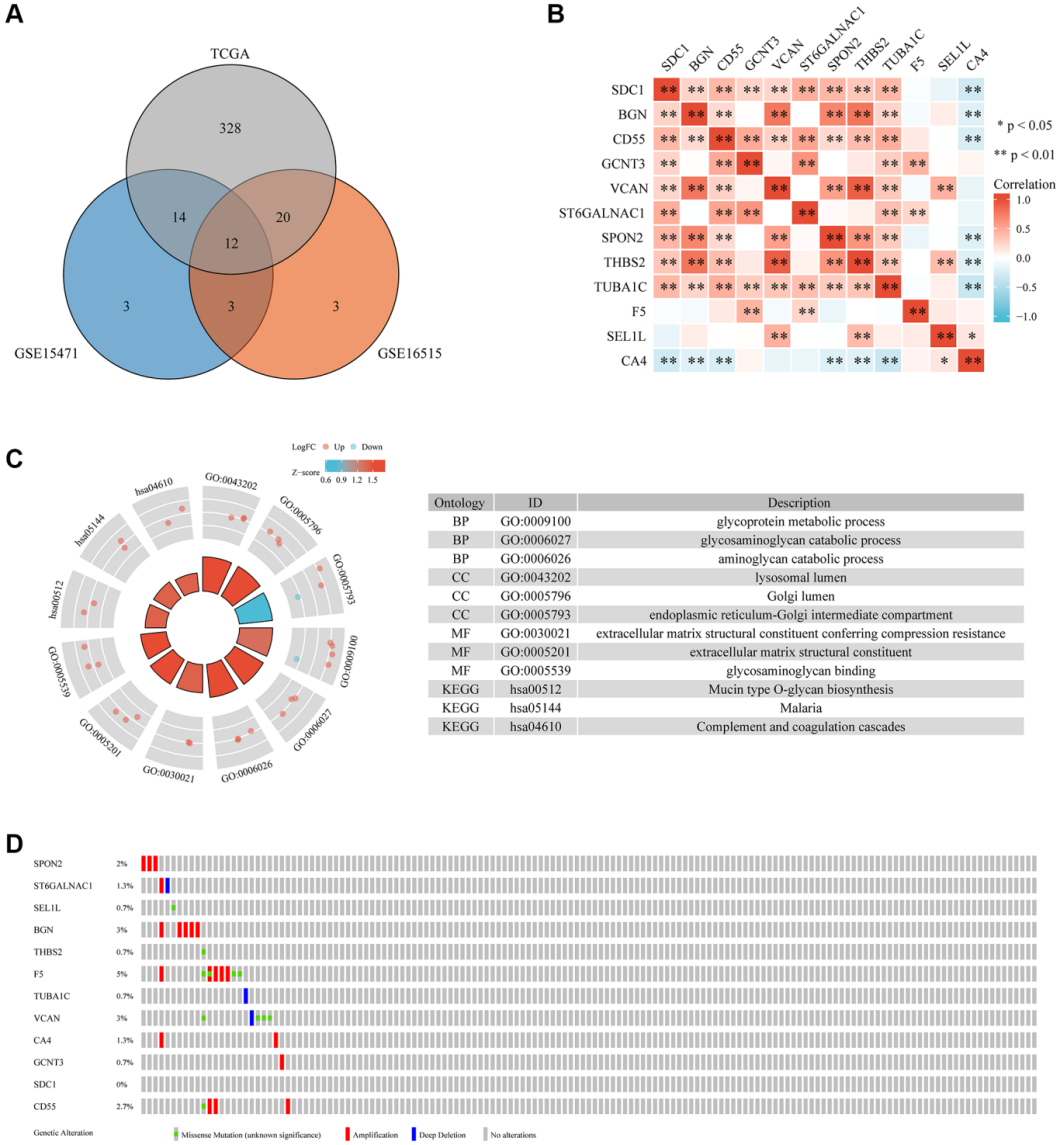
**The differential expression, interaction, functional enrichment, and mutant landscape analysis of GRGs in PC.** (**A**) Genes differentially expressed between TCGA and GEO. (**B**) A heatmap showing the correlations between 12 GRGs. (**C**) GO and KEGG analysis of 12 GRGs in PC. (**D**) Mutant landscape of 12 GRGs.

Differentially expressed glycosylation-related genes (DE-GRGs) were identified in The Cancer Genome Atlas (TCGA)-pancreatic cancer cohort based on the mRNA expression profile in TCGA (Supplementary Table 1). Matching the mRNA sequencing data for differential expression between pancreatic cancer and adjacent tissues using the Gene Expression Omnibus (GEO) database (Supplementary Tables 2 and 3) identified 12 DE-GRGs in pancreatic cancer (Figure 1A). Spearman’s correlation analysis revealed that most DE-GRGs were significantly correlated (Figure 1B). For example, BGN, CD55, GCNT3, VCAN, ST6GALNAC1, SPON2, THBS2, and TUBA1C positively correlated with syndecan-1 (SDC1), while CA4 negatively correlated with SDC1. Gene Ontology (GO) enrichment analysis showed a correlation between the expression of these GRGs and several processes, including glycoprotein metabolism, glycosaminoglycan (GAG) catabolism, aminoglycan catabolism, GAG binding, lysosomal lumen, Golgi lumen, endoplasmic reticulum (ER)-Golgi intermediate compartment, extracellular matrix (ECM) structural constituent conferring compression resistance, and an ECM structural constituent (Figure 1C). Additionally, the Kyoto Encyclopedia of Genes and Genomes (KEGG) pathway analysis revealed that these genes were mainly involved in mucin-type O-glycan biosynthesis, and complement and coagulation cascades. Lastly, analysis of the genetic changes revealed that seven DE-GRGs had a mutation rate of >1%, with the highest mutation rate found in F5 genes (5%). Amplification and missense mutations accounted for most mutations (Figure 1D).

### Determination of pancreatic cancer subtypes using DE-GRGs

**Figure 2:**
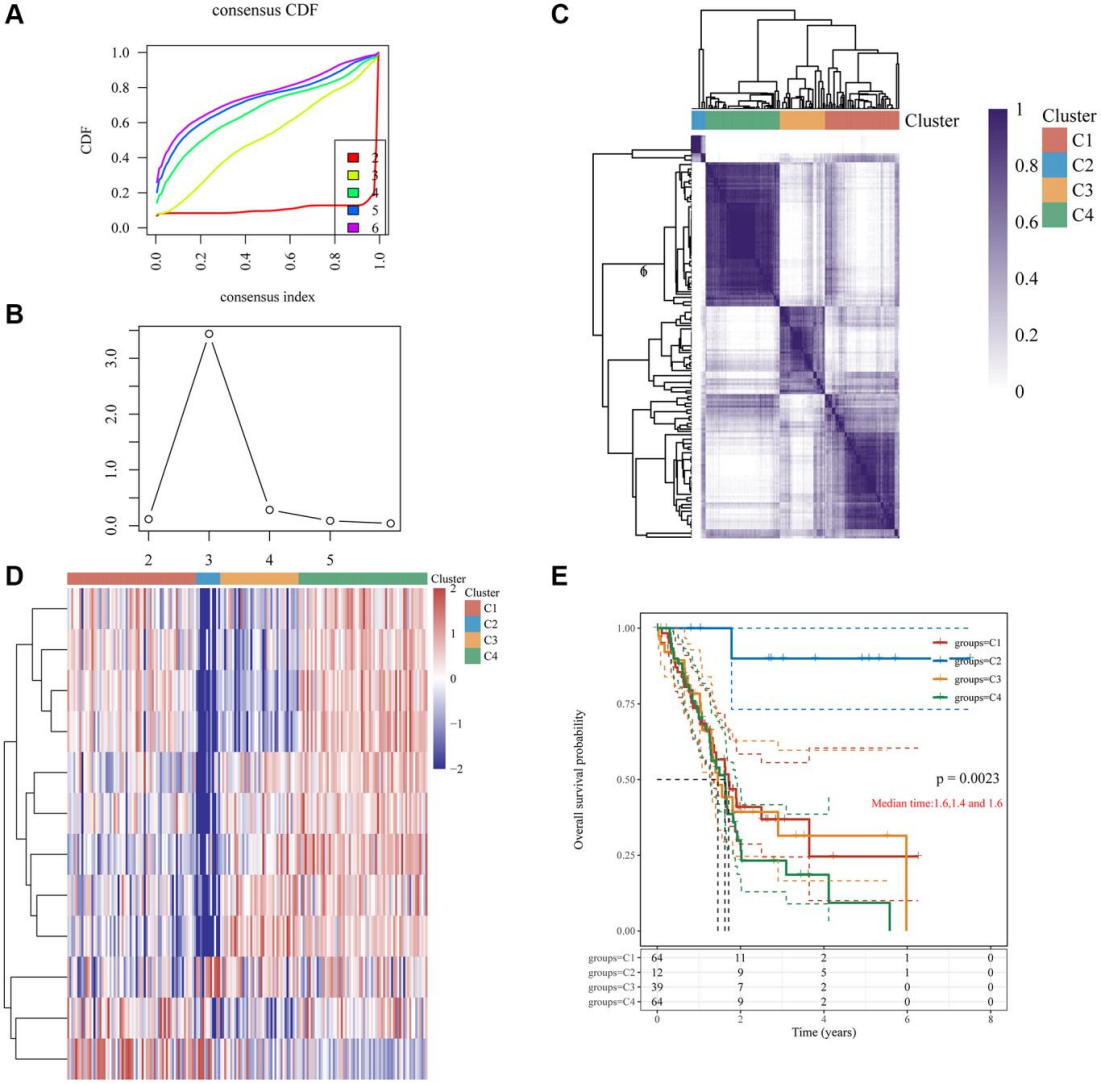
**Clustering of PC molecular subgroups based on 12 GRGs.** (**A**) Cumulative distribution function (CDF) curve. (**B**) CDF delta area curve, which shows how the area under the CDF curve changes between k and k-1 for each category number. The horizontal axis represents the number k, and the vertical axis represents the change in area under the CDF curve; each line represents a relative change. (**C**) A heatmap representing the consensus matrix for k = 4 derived from consensus clustering. Rows and columns in the matrix represent samples, and the degrees of consistency are represented by white to dark blue. (**D**) The heatmap of 12 GRGs in 4 clusters. (**E**) The KM survival curve of different groups in TCGA data sets.

**Figure 3:**
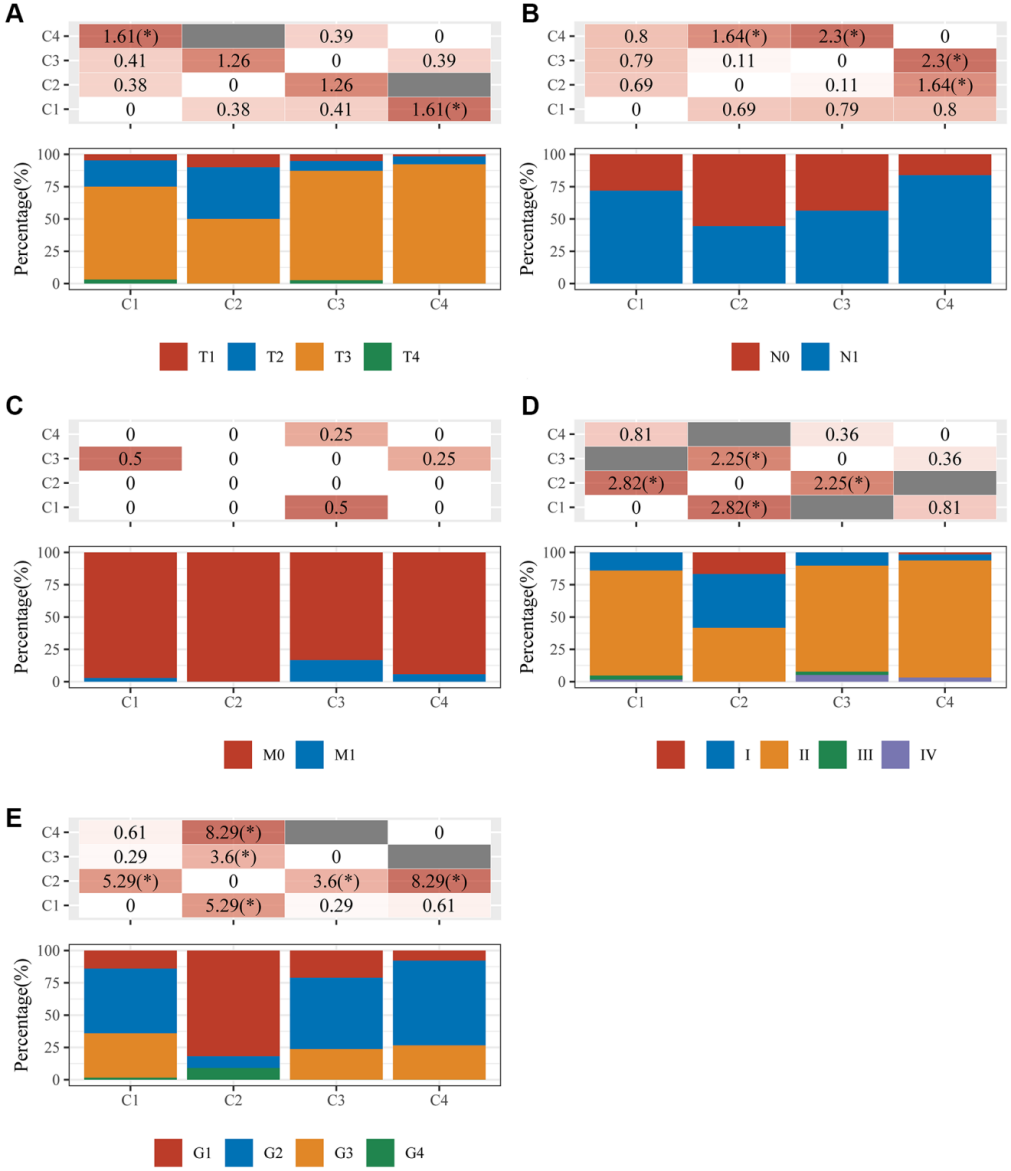
**An analysis of clinical characteristics in four clusters.** The horizontal axis represents the different sample groups. The vertical axis indicates how much clinical information is present in each grouped sample. The table above shows the clinical feature significance *p*-value (-log10) (based on the chi-square test). (**A**) T staging; (**B**) N staging; (**C**) M staging; (**D**) clinical stage; (**E**) clinical grade.

TCGA-pancreatic cancer samples were divided into four clusters (group 1 (C1), group 2 (C2), group 3 (C3), and group 4 (C4)) based on 12 DE-GRGs (Figure 2A–2D). Kaplan–Meier plots showed significant survival disparities between these four groups, group C4 had the worst prognosis and group C2 had the best prognosis (Figure 2E). Further analysis was conducted on the distribution of the tumor, node, and metastasis (TNM) stage and pathological grade among the four subgroups. The samples in cluster-1 showed higher T classifications, but low clinical stage and histological grade; cluster-2 had higher N classification, clinical stage, and pathological grade; cluster-3 had higher N classification, but lower clinical stage and histological grade; and cluster-4 had lower T classification, N classification, clinical stage, and histological grade (Figure 3).

### C1, C2, C3, and C4 immune status and stemness

**Figure 4:**
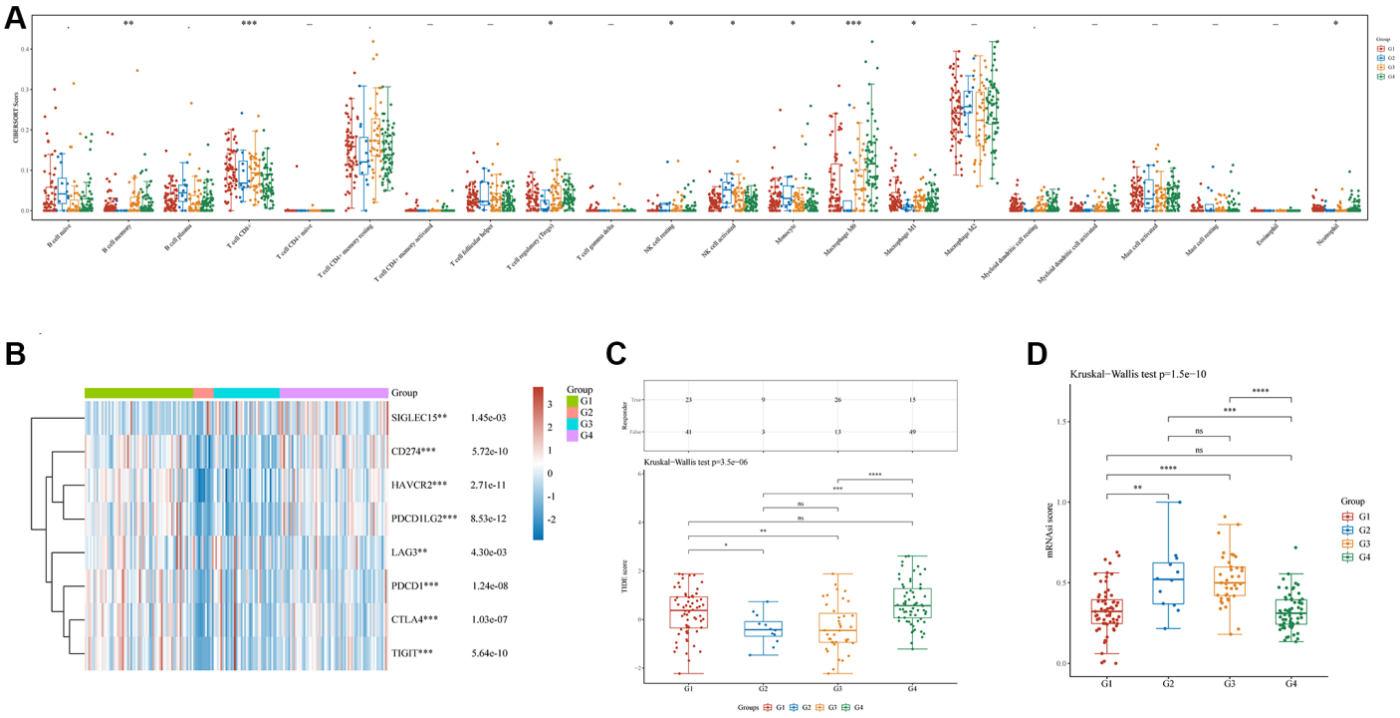
**Immune and stemness analysis in four clusters.** (**A**) Analysis of immune infiltration by C1, C2, C3, and C4 based on the CIBERSORT algorithm; the horizontal axis represents immune cells, while the vertical axis displays immune scores (^*^p < 0.05, ^**^p < 0.01, ^***^p < 0.001). (**B**) Comparison of immune-checkpoint gene expression in C1, C2, C3, and C4; the horizontal axis shows different immune checkpoint genes, while the vertical axis displays the expression level (^*^p < 0.05, ^**^p < 0.01, ^***^p < 0.001). (**C**) A statistical table showing the immune response and the distribution of scores for the different groups, according to the prediction. (**D**) A comparison of stemness for C1, C2, C3, and C4 using mRNAsi scores and the OCLR algorithm.

Immunedeconv (combining CIBERSORT, EPIC, MCP-counter, quanTIseq, TIMER, and xCell software) was used to assess the immune infiltration among these four groups [22]. This analysis used the most widely used algorithm, CIBERSORT [23]. There were significant differences in the counts of CD8+ T cells (*p* < 0.001), regulatory T cells (Tregs) (*p* < 0.05), memory B cells (*p* < 0.01), resting natural killer (NK) cells (*p* < 0.05), activated NK cells (*p* < 0.05), monocytes (*p* < 0.05), M0 macrophages (*p* < 0.001), M1 macrophages (*p* < 0.05), and neutrophils (*p* < 0.05) (Figure 4A). Additionally, the R software tools ggplot2 and pheatmap were used to analyze the four immune checkpoint gene subtypes (ICGs) and significant differences were discovered between them (all *p* < 0.01, Figure 4B). The TIDE algorithm was used to predict cancer immune responses [22]. Clusters C1 and C4 performed better than clusters C2 and C3 (all *p* < 0.05, Figure 4C). This finding indicates that clusters C2 and C3 might achieve more clinical benefit following ICBs. Finally, examination of the mRNA stemness index (mRNAsi) levels of the four subtypes using the one-class logistic regression (OCLR) algorithm [24] revealed significant differences between clusters C1 and C2 (*p* < 0.01), C1 and C3 (*p* < 0.0001), C2 and C4 (*p* < 0.001), and C3 and C4 (*p* < 0.0001) (Figure 4D).

### Development and validation of a glycosylation-related prognostic signature

**Figure 5:**
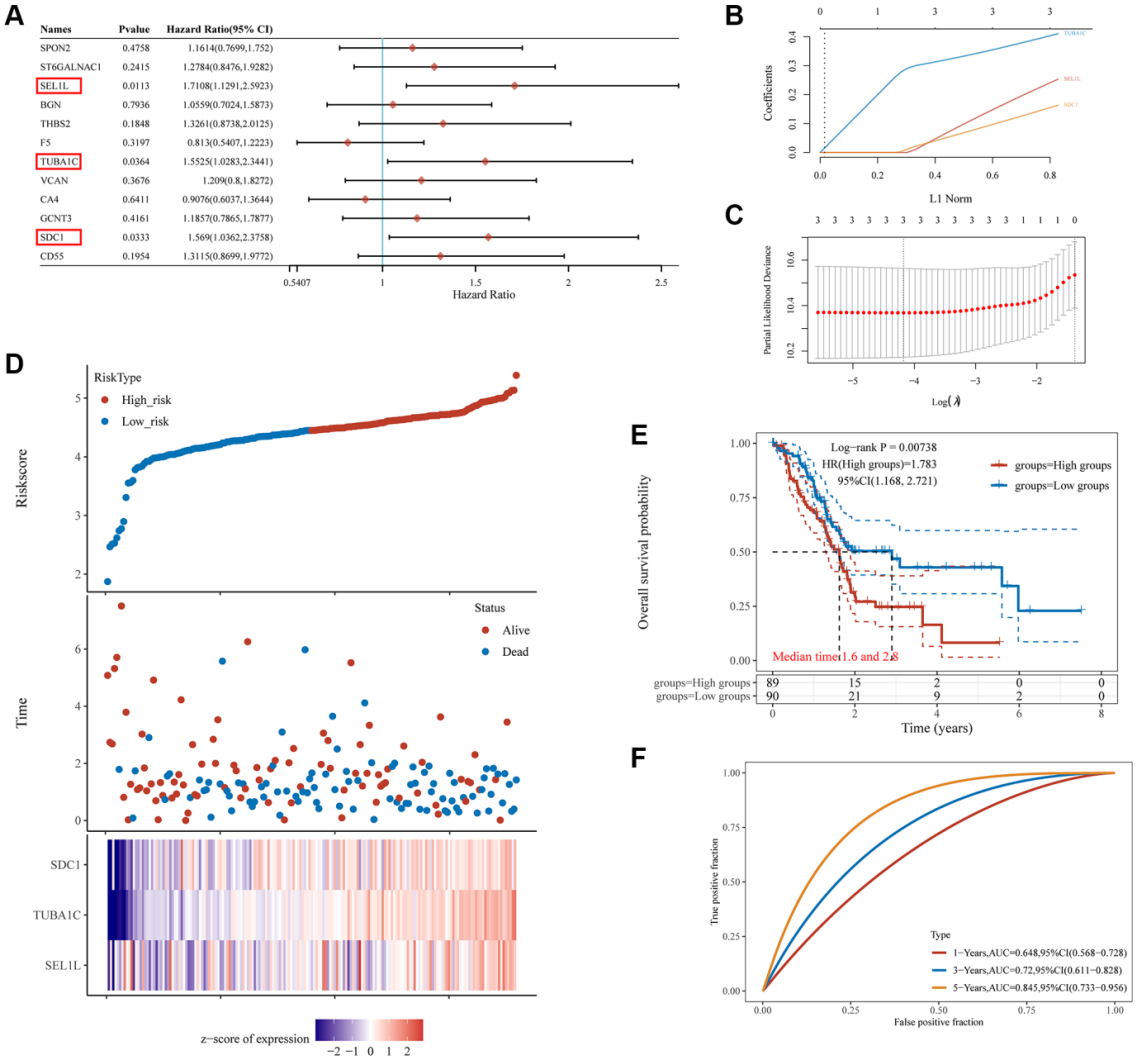
**The prognostic signature of PC based on 3 GRGs in the TCGA database.** (**A**) Log-rank test identifies 3 GRGs correlated to OS in PC patients. (**B**) Lambda parameter shows the coefficients of 3 GRGs. Lambda is represented horizontally, while coefficients are represented vertically. (**C**) The partial likelihood deviance versus log(λ) was calculated using the LASSO Cox regression model. (**D**) The relationship between risk score and living status. Graphs in the middle indicate the risk score, scatter diagrams in the middle, and gene expression heat maps below. (**E**) The KM survival curve of the risk model in the TCGA data set. Several groups were tested by log-rank and HR (high expression), representing the risk factors of high expression versus low expression. (**F**) ROC curves for the risk model and AUCs over various periods (one year, three years, five years). Plots revealed better consistency between the nomogram predictions and actual observations (Figure 7D).

The three DE-GRGs shown by Kaplan–Meier analysis were strongly linked with pancreatic cancer patient survival (Figure 5A). We analyzed the prognostic value of these three genes using the TCGA-pancreatic cancer dataset. The results showed that high expression of these genes was associated with poorer prognosis for patients (Supplementary Figure 1). LASSO Cox regression analysis was used to construct prognostic characteristics based on the three GRGs (Figure 5B, 5C). Risk score = (0.2152) × SEL1L + (0.3895) × TUBA1C + (0.1383) × SDC1. Pancreatic cancer patients were assigned a risk score, which divided them into high- and low-risk categories. The relationship between risk score, survival status, and GRG expression was shown in Figure 5D. High-risk scores were inversely connected with survival and positively correlated with risk of death. High-risk pancreatic cancer patients had considerably lower survival periods than low-risk pancreatic cancer patients (Figure 5E), and this prognostic model accurately predicted 1-, 3-, and 5-year survival rates with areas under the receiver operator characteristic (ROC) curves of 0.648, 0.72, and 0.845, respectively (Figure 5F).

**Figure 6:**
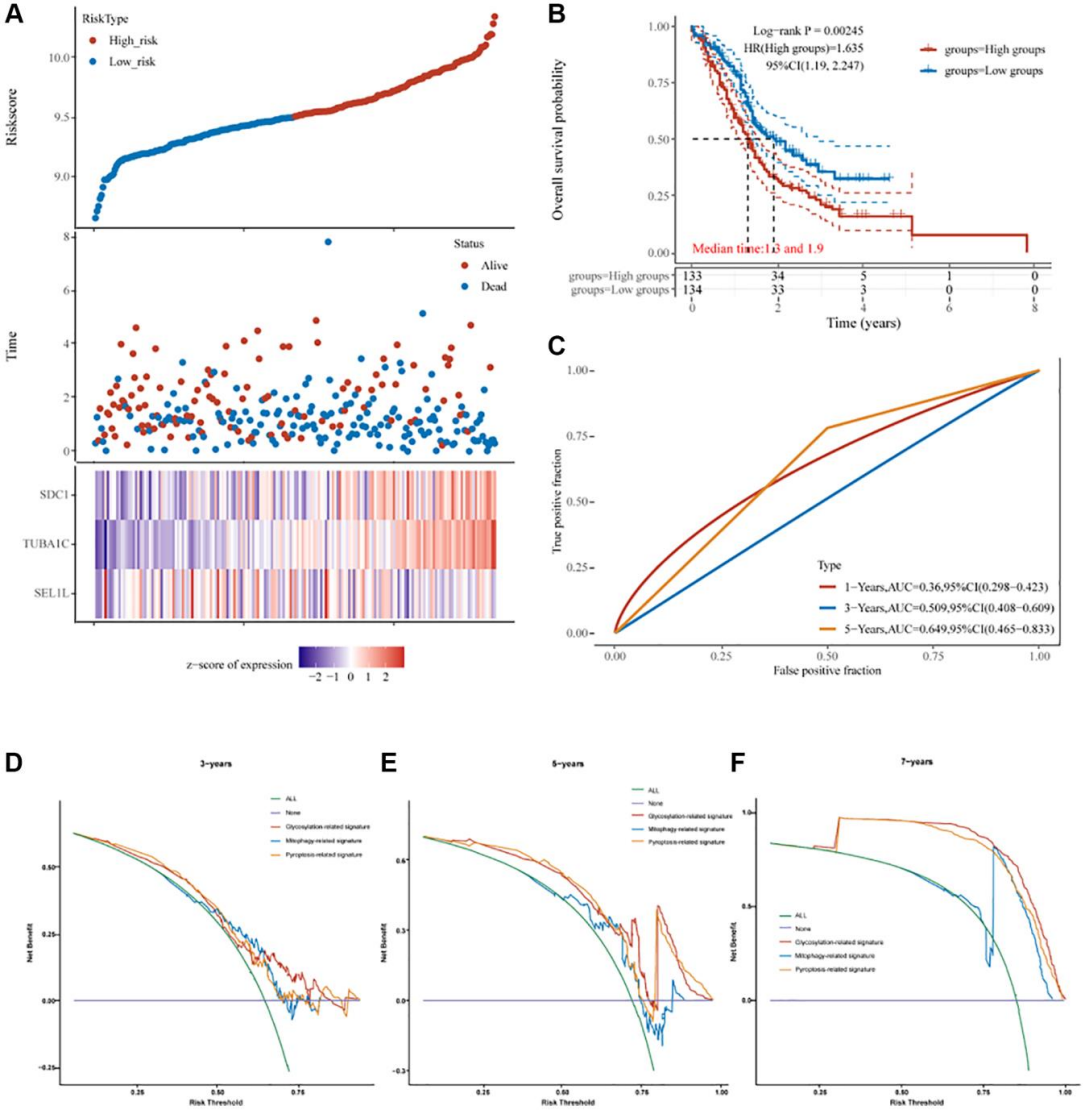
**The prognostic signature of PC based on 3 GRGs in the ICGC database.** (**A**) Lambda parameter shows the coefficients of 3 GRGs. (**B**) The partial likelihood deviance versus log(λ) was calculated using the LASSO Cox regression model. (**C**) The relationship between risk score and living status. (**D**) The KM survival curve of the risk model in the ICGC data set. (**E**) ROC curves for the risk model and AUCs over various periods (1 year, 3 years, 5 years). (**F**) Comparisons of 3 prognostic signatures in PC through DCA curve.

The predictive utility of this signature underwent additional validation utilizing pancreatic cancer samples from the International Cancer Genome Collaboration (ICGC) database (Figure 6A, 6B). Risk scores were used to classify pancreatic cancer patients as high- or low-risk in line with the above results (Figure 6C). The survival rate was lower in the high-risk group compared to that in the low-risk group (*p* < 0.01, HR = 1.635, Figure 6D). The area under the curves (AUCs) were 0.36, 0.509, and 0.649 for 1, 3, and 5 years of survival, respectively, based on the three-gene model (Figure 6E). In parallel, the glycosylation-related signature was compared with other prognostic models for pancreatic cancer [25, 26]. Our prognostic signature showed a higher prognostic predictive value than a mitophagy-related signature and pyroptosis-related signature based on the 3-, 5-, and 7-year decision curve analyses (DCA) (Figure 6F).

### A prognostic nomogram that uses prognostic signatures from TCGA to predict overall survival in pancreatic cancer cohorts

**Figure 7:**
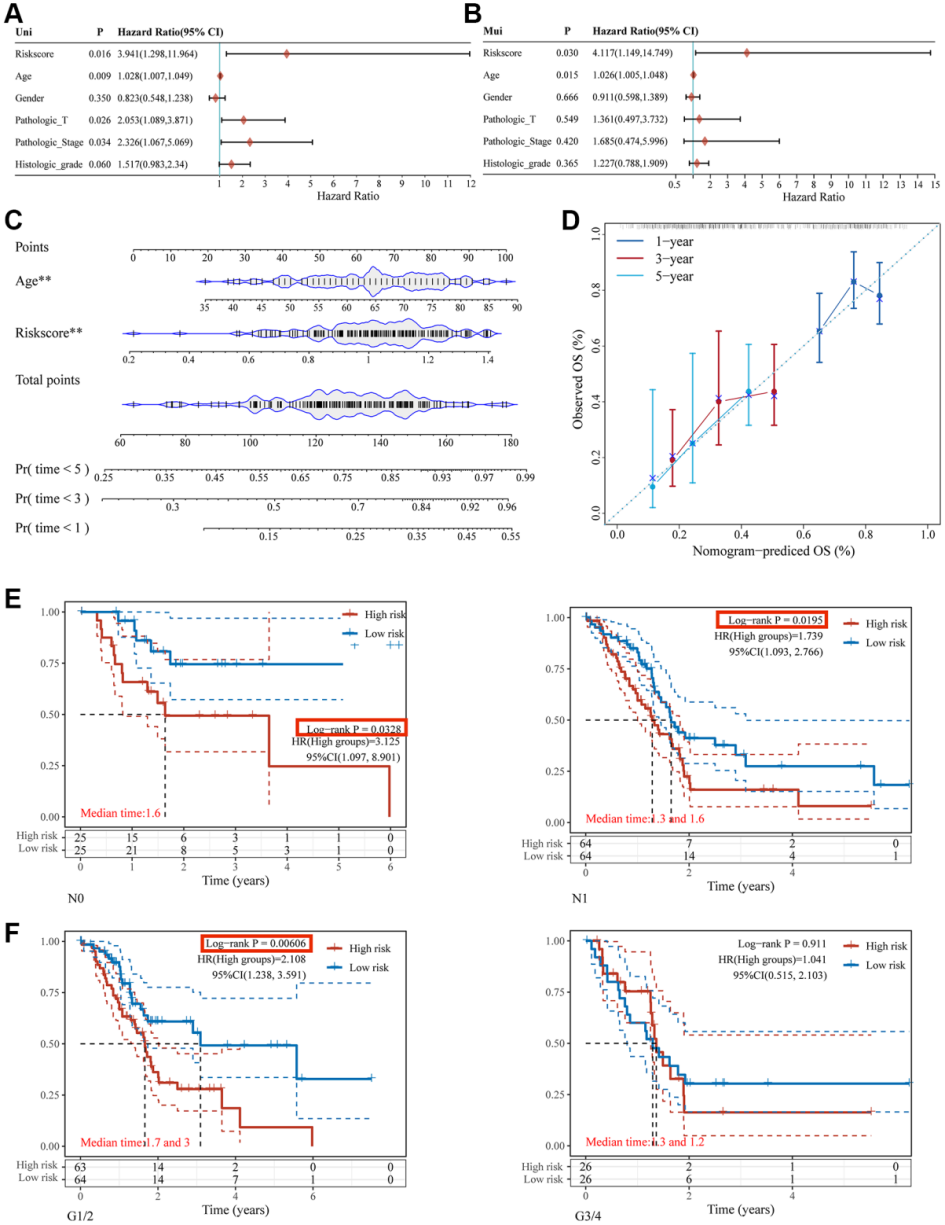
**Nomogram construction for patients with PC in the TCGA database.** The forest plot of the univariate (**A**) and multivariate (**B**) association between risk scores and clinicopathological characteristics. (**C**) A nomogram based on two independent prognostic factors was developed to predict the OS in patients with PC over 1, 3, and 5 years. (**D**) The calibration plot for internal validation of the nomogram. Survival analyses using the glycosylation-related signature in patients with different N staging (**E**) and clinical grade (**F**).

Age and risk-score based prognostic signatures were independent risk factors for OS of pancreatic cancer using univariate and multivariate Cox regression analyses (*p* < 0.05) (Figure 7A, 7B). These two factors were integrated to develop a nomogram for overall survival prediction (Figure 7C). The regression coefficients for each influencing factor were added to obtain the total score. The overall score was converted to the probability that each outcome would occur to calculate the predicted value of each outcome. There was a better correlation between the nomogram predictions and actual survival rates for the first, third, and fifth years based on calibration plots (Figure 7D).

### Relationship between the glycosylation-related signature and clinical features

The glycosylation-related signature was evaluated in stratified cohorts of patients with pancreatic cancer according to smoking history (yes or no), drinking history (yes or no), grade (G1/2 or G3/4), T stage (T1/2 or T3/4), N stage (N0 or N1), and radiotherapy (yes or no). Kaplan–Meier curves showed that the high-risk group exhibited a shorter OS than the low-risk group in no-smoking (Supplementary Figure 2A), drinking (Supplementary Figure 2B), no-radiotherapy (Supplementary Figure 2C), T1/2 staging (Supplementary Figure 2D), N0/1 staging (Figure 7E), and G1/2 (Figure 7F) groups. This indicated that this prognostic signature should be used with consideration for the effects of clinical factors.

### Glycosylation-related signature to predict immune infiltration

**Figure 8:**
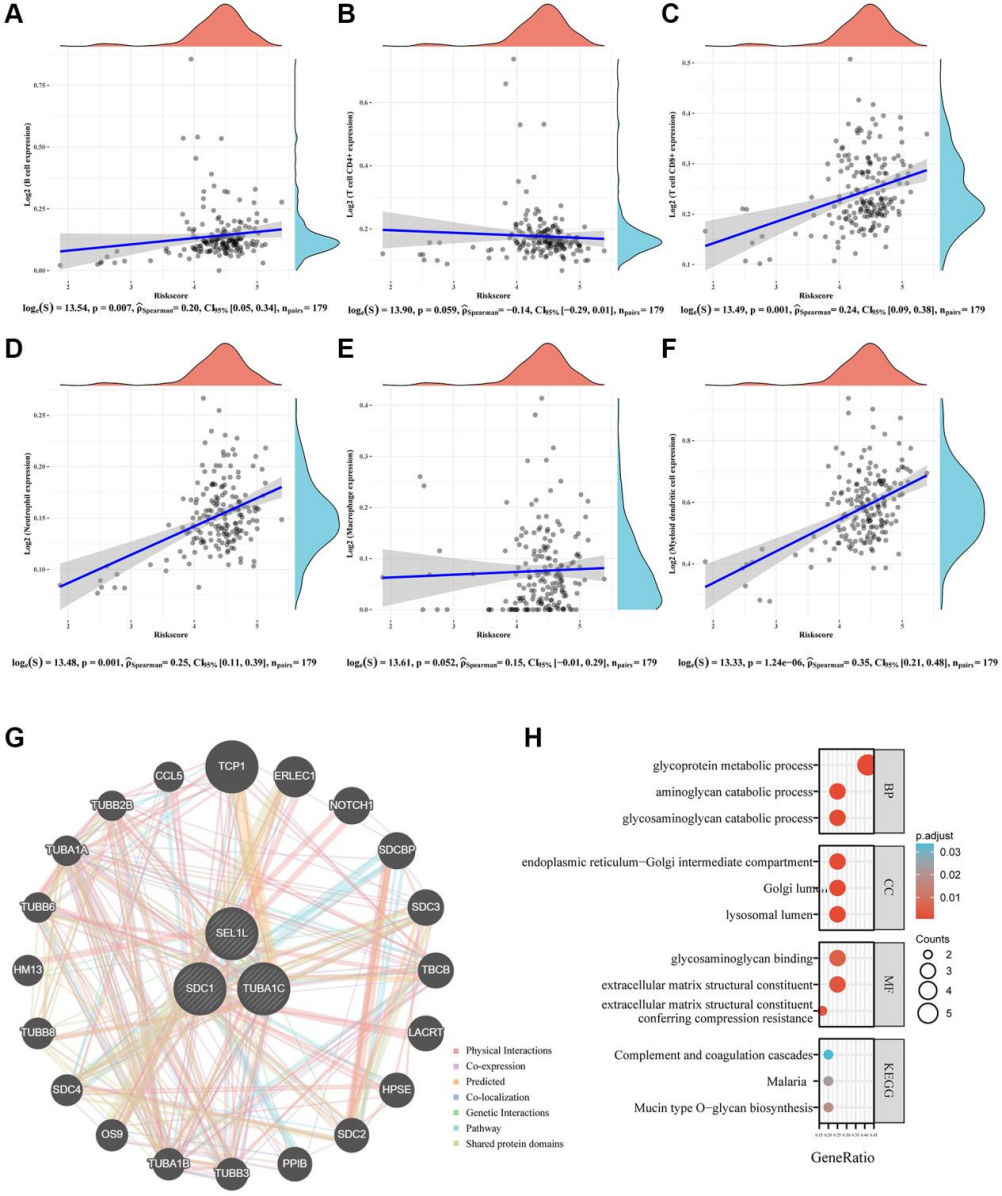
**Immune status and biological functions of glycosylation-related signature.** (**A**–**F**) The relationship between the abundance of six kinds of immune cells (B cells, CD4+T cell, CD8+T cell, neutrophil, macrophage, and dendritic cell) and the risk score of the glycosylation-related signature. (**G**) A regulatory network consisting of eight genes and 20 potential binding proteins was constructed using the GeneMANIA database. (**H**) GO and KEGG analyses for 28 genes.

The process by which immune cells enter tumor tissues via circulation is known as tumor immune cell infiltration. Tumor-associated immune cells may serve as targets for drugs that can improve survival rates because they are linked to clinical outcomes [27]. This study analyzed whether a glycosylation-related signature derived from GRGs was related to immune infiltration. The glycosylation-related signature was positively associated with B cells (Figure 8A), CD8+T cells (Figure 8C), neutrophils (Figure 8D), and myeloid dendritic cells (Figure 8F), but not with CD4+T cells (Figure 8B) and macrophages (Figure 8E). Hence, we conclude that patients with high GRG expression may have a poorer prognosis owing to tumor immune infiltration.

### The biological functions and pathways of the glycosylation-related signature

GeneMANIA was used to construct an interactive network to investigate whether SEL1L, TUBA1C and SDC1 were related. Twenty additional binding partners were identified (Figure 8G). Next, GO and KEGG to analysis were used for pathway enrichment of 23 genes. GO analysis showed that these 23 genes were mainly involved in GAG catabolic processes, aminoglycan catabolic processes, GAG metabolic processes, microtubules, vacuolar lumen, lysosomal lumen, structural constituent of the cytoskeleton, GTPase activity, and GTP binding (Figure 8H). Moreover, the majority of these 23 genes were engaged in prion disease, gap junctions, and phagosomes according to KEGG pathway analysis (Figure 8H).

### The relationship between SEL1L, TUBA1C, and SDC1 expression and clinical features

**Figure 9:**
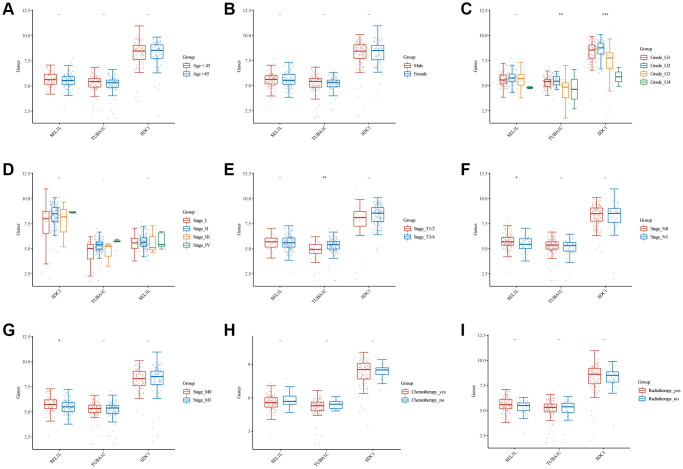
**Comparisons of clinical characteristics between 3 GRGs.** Age (**A**), gender (**B**), clinical grade (**C**), clinical stage (**D**), T staging (**E**), N staging (**F**), M staging (**G**), chemotherapy history (**H**), and radiotherapy history (**I**) of high and low SEL1L/TUBA1C/SDC1 expression groups.

As mentioned above, glycosylation-related signatures based on three GRGs (SEL1L, TUBA1C, and SDC1) were affected by various clinical features. We classified pancreatic cancer patients into subgroups based on age (<45 or >45), sex (male or female), grade (G1, 2, 3, or 4), T stage (T1/2 or T3/4), N staging (N0 or N1), M stage (M0 or M1), clinical stage (I, II, III, or IV), chemotherapy (yes or no), and radiotherapy (yes or no), and compared the expression levels of SEL1L/TUBA1C/SDC1 between them. There was an insignificant correlation between the GRGs and age (Figure 9A) or sex (Figure 9B). High expression of TUBA1C and SDC1 was negatively correlated with grade (Figure 9C). No significant results were observed between the GRGs and clinical stage (Figure 9D). High TUBA1C expression positively correlated with T staging (Figure 9E), and N staging (Figure 9F) and M staging (Figure 9G) were adversely linked with high expression of SEL1L. Similarly, there was no correlation between the expression of GRGs and chemotherapy (Figure 9H) or radiotherapy (Figure 9I).

### The biological significance of SEL1L/TUBA1C/SDC1 in pancreatic cancer

TCGA-pancreatic cancer data were classified according to NRG expression levels, SEL1L, TUBA1C, and SDC1, and each group was analyzed using GO and KEGG. The cutoff was set so that the top 50% represented high expression of SDC1, SEL1L, and TUBA1C and the bottom 50% represented low expression of these genes.

**Figure 10:**
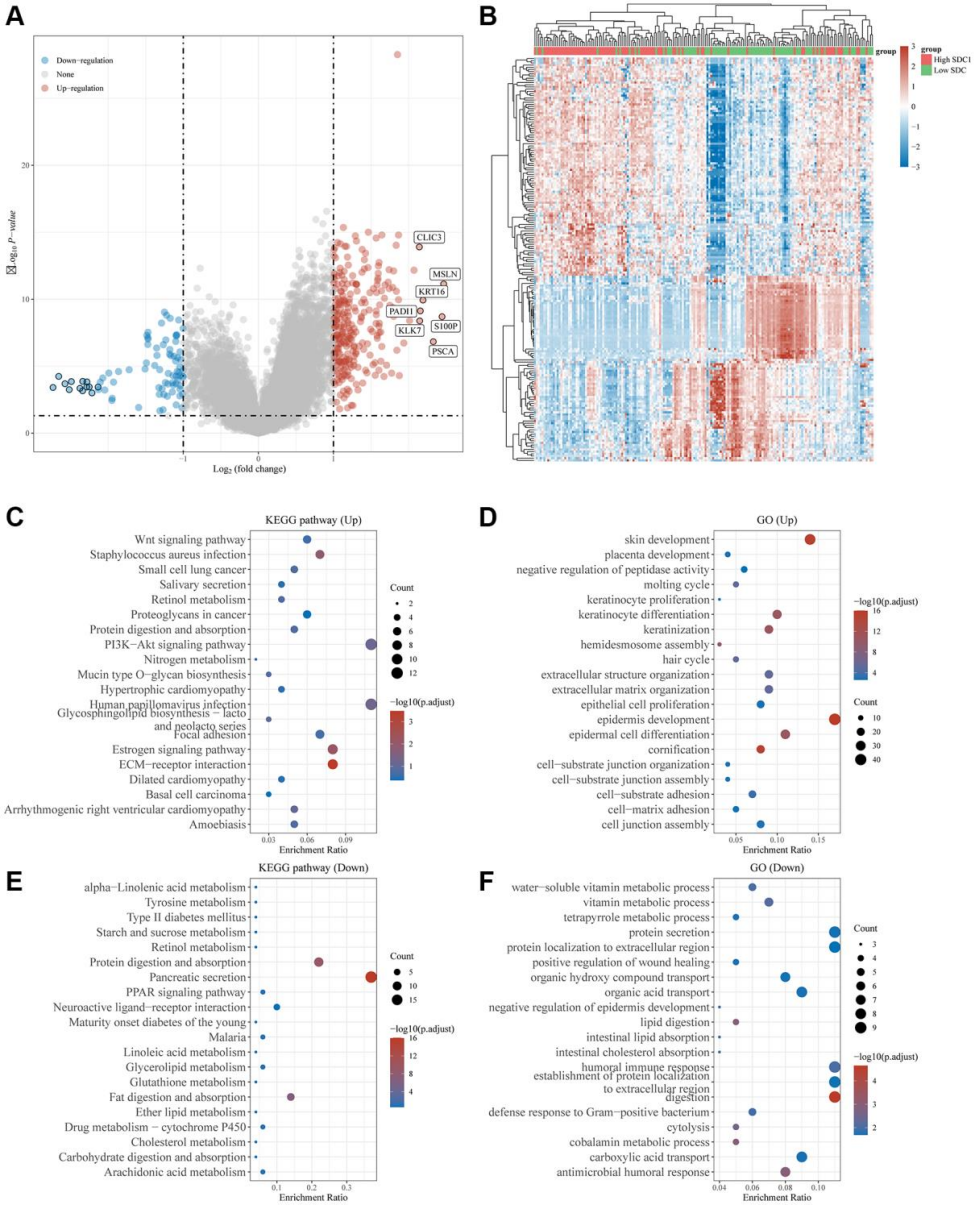
**Differential expression and enrichment analysis of high and low SDC1 expression groups.** (**A**) Volcano plots show the differential gene expression between SDC1 high expression and SDC1 low expression groups using fold-change values and adjusted *p*-values. (**B**) Heatmap showing differential gene expression (only 50 genes were displayed due to a large number of genes). (**C**–**F**) KEGG and GO analyses revealed the signaling pathways associated with up-regulated and down-regulated genes in the SDC1 high/low expressed groups. An enriched pathway is considered when *p* < 0.05 or FDR <0.05.

High expression of SDC1 in TCGA-pancreatic cancer cohort resulted in 269 upregulated genes and 85 downregulated genes (Figure 10A, 10B). The 269 upregulated genes were associated with pancreatic physiological functions (including epidermal development, keratinocyte differentiation, and ECM organization) and activation processes such as the PI3K-Akt signaling pathway, Wnt signaling pathway, and ECM receptor interaction (Figure 10C, 10D). The 85 downregulated genes were mainly involved in physiological processes, including protein secretion, vitamin metabolic process, and humoral immune response, together with activation processes, such as the PPAR signaling pathway and protein digestion (Figure 10E, 10F).

High expression of TUBA1C in TCGA-pancreatic cancer cohort included 143 upregulated genes and 42 downregulated genes (Supplementary Figure 3A, 3B). The 143 upregulated genes were associated with pancreatic physiological functions (including epidermal development, keratinocyte differentiation, and ECM organization) and activation processes, such as PI3K-Akt signaling, the estrogen signaling pathway, and ECM receptor interaction (Supplementary Figure 3C, 3D). The 42 downregulated genes were mainly involved in physiological processes, including negative regulation of proteolysis, vitamin metabolic processes, and humoral immune responses (Supplementary Figure 3E, 3F).

High expression of SEL1L in TCGA-pancreatic cancer cohort included 48 upregulated genes and 7 downregulated genes (Supplementary Figure 4A, 4B). The 48 upregulated genes were associated with pancreatic physiological functions (including epidermal development, positive regulation of keratinocyte differentiation, and ECM organization) and activation processes such as pancreatic secretion, protein digestion and absorption, and fat digestion and absorption (Supplementary Figure 4C, 4D).

### Correlation between SEL1L/TUBA1C/SDC1 expression, immune status, and stemness

**Figure 11:**
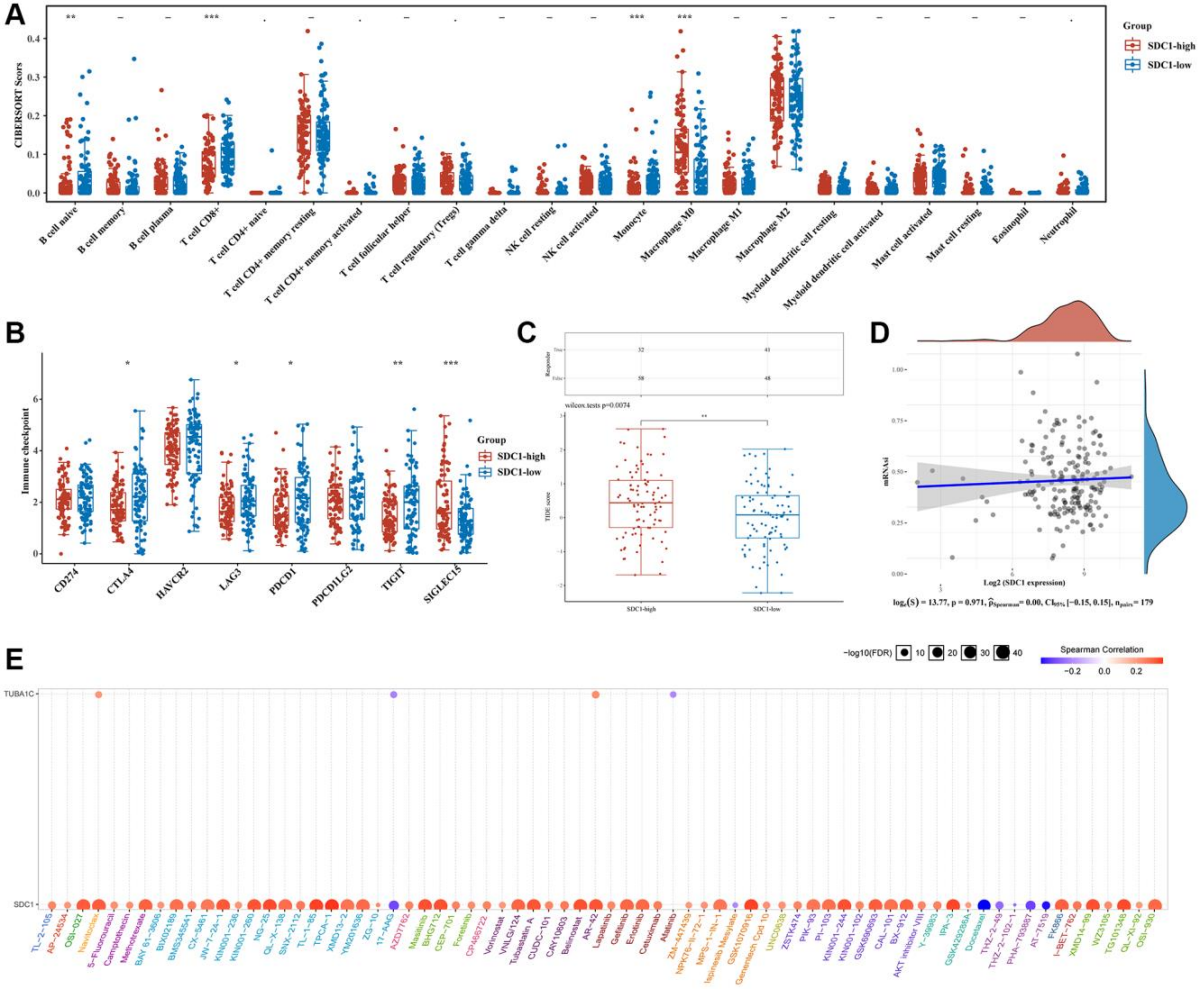
**Comparison of immune status and stemness between groups that express high and low levels of SDC1.** (**A**) An analysis of immune infiltration was obtained using the CIBERSORT algorithm for the high expression group of SDC1 and the low expression group of SDC1; the horizontal axis represents immune cells, while the vertical axis displays immune scores (^*^p < 0.05, ^**^p < 0.01, ^***^p < 0.001). (**B**) Comparison of immune-checkpoint gene expression in SDC1 high expression group and SDC1 low expression group. (**C**) Table showing immune response statistics and immune response scores for different groups. (**D**) With the OCLR algorithm, SDC1 low expression, and SDC1 high expression groups were compared for stemness. (**E**) The correlation between SDC1/TUBA1C and GDSC drug sensitivity in PC.

Immune infiltration data from the high- and low-expression SEL1L, TUBA1C, and SDC1 pancreatic cancer groups were gathered using CIBERSORT algorithms. High levels of SDC1 expression positively correlated with naive B cells (*p* < 0.01), CD8+ T cells (*p* < 0.001), and monocytes (*p* < 0.001), and poorly correlated with M0 macrophages (*p* < 0.001) (Figure 11A). High SEL1L levels positively correlated with M1 macrophages (*p* < 0.05), M2 macrophages (*p* < 0.05), resting myeloid dendritic cells (*p* < 0.01), activated mast cells (*p* < 0.01), and neutrophils (*p* < 0.01), and poorly correlated with memory B cells (*p* < 0.05), follicular helper T cells (*p* < 0.01), and Tregs (*p* < 0.001) (Supplementary Figure 5A). High TUBA1C levels positively correlated with M0 macrophages (*p* < 0.05) and M1 macrophages (*p* < 0.05), but poorly correlated with naive B cells (*p* < 0.05) and CD8+ T cells (*p* < 0.05) (Supplementary Figure 5E).

Next, we examined the relationship between ICGs and SEL1L, TUBA1C, and SDC1. SDC1 expression strongly correlated with SIGLEC15 (*p* < 0.001) and poorly correlated with CTLA4 (*p* < 0.05), LAG3 (*p* < 0.05), PDCD1 (*p* < 0.05), and TIGIT (*p* < 0.01) (Figure 11B). SEL1L expression positively correlated with three out of eight ICGs including CD274 (*p* < 0.001), HAVCR2 (*p* < 0.01), and PDCD1LG2 (*p* < 0.001) (Supplementary Figure 5B). TUBA1C expression strongly correlated with CD274 (*p* < 0.05) and SIGLEC15 (*p* < 0.01) (Supplementary Figure 5F). The TIDE algorithms revealed that high SDC1 expression correlated with a poor immune response (Figure 11C), whereas the expression of SEL1L and TUBA1C was not closely related to the immune response (Supplementary Figure 5C and 5G). There was no crucial correlation between stemness and the expression of SDC1 (Figure 11D), SEL1L (Supplementary Figure 5D), or TUBA1C using the OCLR algorithms (Supplementary Figure 5H).

### Drug-sensitivity analysis of SEL1L/TUBA1C/SDC1 in pancreatic cancer

Gene expression-drug correlation analysis is necessary to develop a therapeutic target. Drug-sensitivity analysis showed that the expression of SDC1 and TUBAC1 was positively correlated with some or most medications (Figure 11E). Remarkably, high SDC1 expression was significantly associated with nearly all the existing drugs. This indicated that SDC1 might be a possible therapeutic target for pancreatic cancer.

### Validating the expression of SEL1L/TUBA1C/SDC1 *in vitro* and *in vivo*

**Figure 12:**
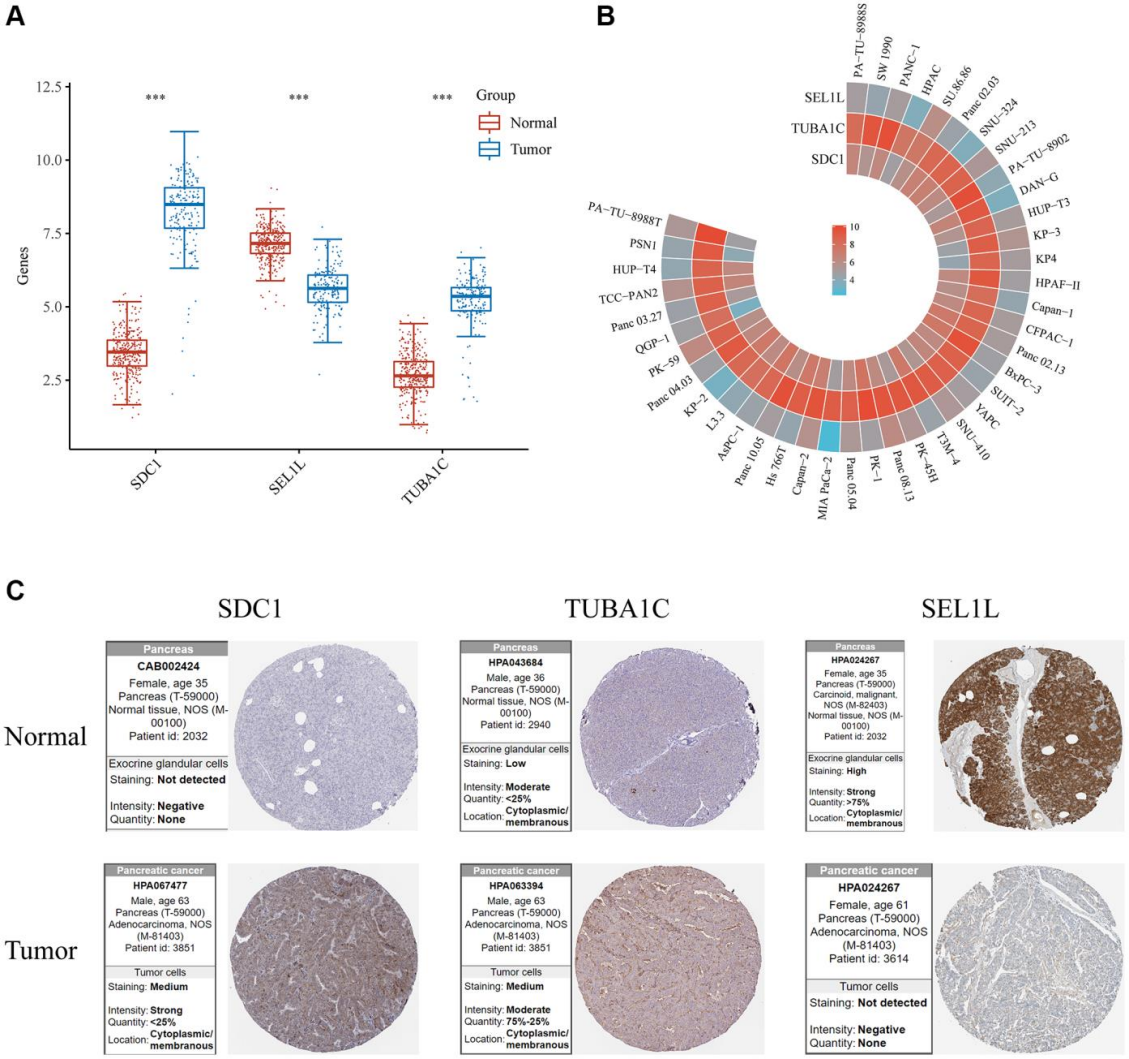
**Validation of the mRNA/protein expression levels of SDC1/SEL1L/TUBA1C *in vitro* and *in vivo*.** (**A**) The mRNA expression levels of SDC1/SEL1L/TUBA1C of PC and normal pancreas in TCGA database. (**B**) The mRNA expression levels of SDC1/SEL1L/TUBA1C of PC cell lines in the CCLE database. (**C**) The protein expression levels of SDC1/SEL1L/TUBA1C of PC and normal pancreas in the HPA database.

The SEL1L/TUBA1C/SDC1 mRNA expression levels were compared in normal and pancreatic cancer tissues using TCGA. Pancreatic cancer samples had a higher expression of TUBA1C and SDC1 compared with the standard samples, while SEL1L expression was lower *in vitro* and *in vivo* (Figure 12A). A comparison of SEL1L/TUBA1C/SDC1 mRNA expression levels in various pancreatic cancer cell lines using the Cancer Cell Line Encyclopedia (CCLE) database showed that most pancreatic cancer cells expressed less SEL1L than SDC1 or TUBA1C (Figure 12B). Additionally, we compared the levels of SEL1L/TUBA1C/SDC1 expression in normal and pancreatic cancer tissues using the Human Protein Atlas (HPA) database. In the HPA database, the expression levels of TUBA1C, SDC1, and SEL1L displayed a comparable pattern (Figure 12C).

### Validation of the glycosylation-related signature by qRT-PCR

**Figure 13:**
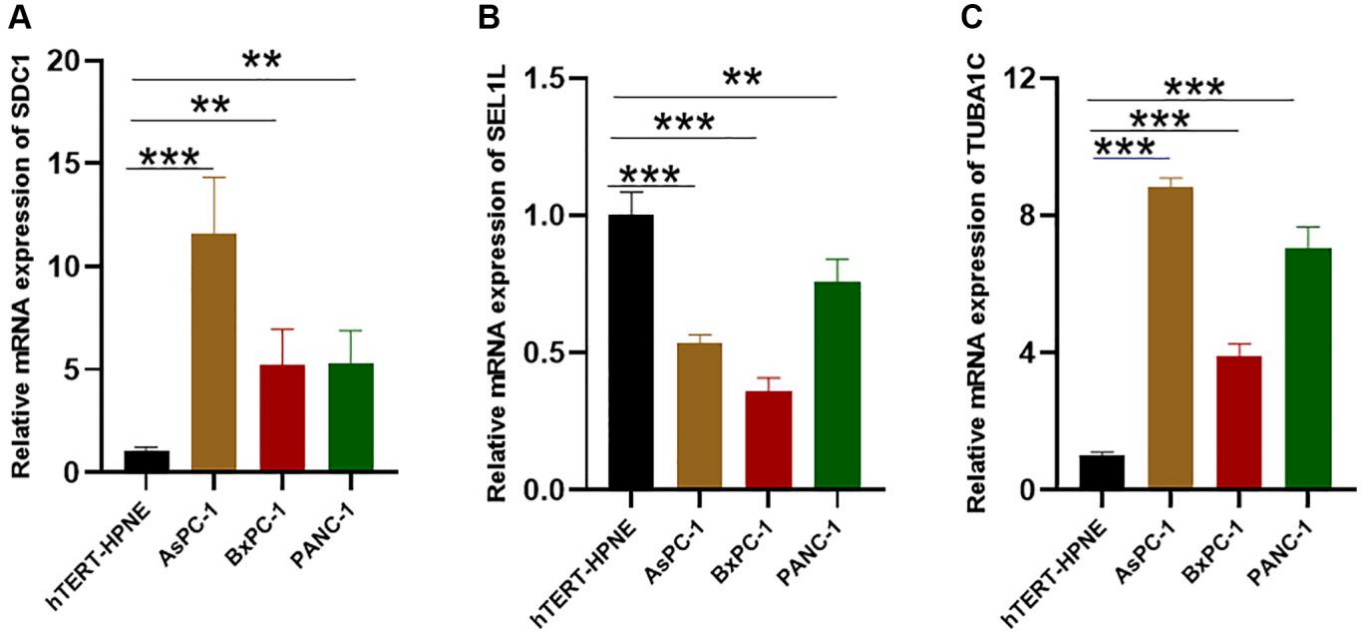
**The expression of the glycosylation-related signature in cell lines.** (**A**–**C**) qRT-PCR results of the glycosylation-related signature in PC cell lines (AsPC-1, BxPC-3, PANC-1) and control cell lines (hTERT-HPNE). ^*^p < 0.05, ^**^p < 0.01, ^***^p < 0.001.

In order to further validate 3 GRGs expression in the lab, qRT-PCR in normal pancreatic cells (hTERT-HPNE) and 3 pancreatic cancer cell lines (AsPC-1, BxPC-3, PANC-1) were carried out. The mRNA expression levels of SDC1 and TUBA1C were significantly increased and the expression of SEL1L was relatively lower in PC cell lines compared to hTERT-HPNE (Figure 13A–13C), which were consistent with our bioinformatics analysis results.

## DISCUSSION

Protein glycosylation is the most common post-translational modification that serves diverse biological functions [28]. Almost all proteins are glycosylated in at least one way during their synthesis [29]. A new hallmark of cancer is aberrant glycosylation, which plays a critical role in tumor biology [11]. This may offer an opportunity to predict and treat cancer outcomes. Glycosylation-related gene-based signatures predicted the prognosis of ovarian cancer and head and neck squamous cell carcinoma [30, 31]. However, the role of GRGs in pancreatic cancer is poorly studied; therefore, this study determined their correlation.

Precision medicine is a new approach for obtaining and integrating information from biomedical research and clinical practice [32]. The higher success rates of precision medicine in treating HER2-positive breast cancer [33] and EGFR-positive lung cancer [34] highlight its potential to widely change clinical practice. Our study divided samples into four clusters (C1, C2, C3, and C4) based on the DE-GRG dataset of pancreatic cancer, and examined the differences between the clusters. Survival probability was significantly different between these four clusters using Kaplan–Meier plots: C4 and C2 had the worst and best prognosis, respectively. Immune infiltration and ICG analysis of the four groups revealed significant differences in the immune microenvironment. TIDE analysis confirmed differences in immune responses among the four clusters. These findings show that glycosylation is closely associated with pancreatic cancer and highlight its prognostic value.

We identified three GRGs closely related to pancreatic cancer: SEL1L, TUBA1C, and SDC1. Syndecan 1 (SDC1) is a transmembrane heparan sulfate proteoglycan component that has a variety of functions in the development, proliferation, adhesion, and angiogenesis of tumor cells [35, 36]. In general, SDC1 is downregulated in gastrointestinal cancers. Loss of epithelial SDC1 is associated with poor prognosis in hepatocellular carcinoma, colorectal cancer, and gastric cancer patients, together with high tumor volume and high histological grade [37, 38]. Nevertheless, SDC1 levels are solely elevated in pancreatic cancer (the only gastrointestinal malignancy) which speeds up tumor growth [39]. Serum SDC1 is a promising new biomarker for patients with pancreatic cancer [40]. Suppressor Enhancer Lin12 1-like (SEL1L) is a single-transmembrane protein that resides in the ER and destroys misfolded proteins [41–44]. The SEL1L variant genotype rs12435998 contributes to survival time in pancreatic cancer patients who have received combined chemotherapy and pancreaticoduodenectomy [45]. SEL1L expression reduces pancreatic carcinoma cell aggressiveness *in vivo* and *in vitro* [46]. Moreover, SEL1 regulates the adhesion and proliferation of β-cells through interaction with β1 integrin signaling [47]. Tubulin Alpha 1c (TUBA1C) is a subtype of α-tubulin that is highly correlated with microtubule structure that is essential for cell division and mitosis [48, 49]. Moreover, TUBA1C plays a vital role in cell cycle signaling pathways [50] and can significantly affect tumor growth and progression [51, 52]. Patients with pancreatic cancer have shorter OS when TUBA1C is overexpressed [53]. The findings of our study on SDC1/TUBAC/SEL1L are consistent with those of previous studies.

We created a three-gene signature that precisely predicts the prognosis of individuals with pancreatic cancer on the basis of these three GRGs using data from TCGA and ICGC cohorts. Both datasets showed that low-risk patients had a superior OS compared to high-risk patients. A recent study predicted the prognosis of pancreatic cancer using a two-gene signature (CASP4 and NLRP1) based on genes related to pyroptosis [25]. Additionally, the usefulness of a three-gene signature (PRKN, SRC and VDAC1) was evaluated based on genes related to mitophagy to predict survival in pancreatic cancer patients [26]. Decision curve analysis revealed that the proposed model provided better clinical benefits. Furthermore, our study developed independent prognostic indicators (age scores and risk scores) and a nomogram to accurately predict 1-, 3-, and 5-year survival rates. This may help improve individualized treatment strategies for patients with pancreatic cancer. These results showed that glycosylation-related signatures were significantly associated with pancreatic cancer patient prognosis and were better predictors of pancreatic cancer outcomes.

Recent advances in immunotherapy have led to notable improvements in the treatment of a wide variety of cancers [54]. Thus, we looked into the potential effects of signals related to glycosylation in the immunological microenvironment. High-risk patients in TCGA cohort had immunological ratings that were noticeably greater than those of low-risk patients. Higher immune scores are linked to poorer prognosis in patients with pancreatic cancer [55, 56]. Correlations between the signature and the immune landscape indicated that this signature could help predict the number of CD8+ T cells, B cells, neutrophils, and myeloid dendritic cells. This might explain why high-risk patients had a poor prognosis.

Drug-sensitivity analysis using the Genomics of Drug Sensitivity in Cancer (GDSC) database showed that SDC1 expression was closely related to existing drugs. Interestingly, a previous study found that SDC1+ cell lines were susceptible to indatuximab ravtansine in breast cancer [57]. Hepatocellular carcinoma cell lines are platinum-resistant when syndecan-1 (SDC1) is upregulated [58]. Our results indicate that SDC1 may be a therapeutic target for the treatment of pancreatic cancer.

This study had a few limitations. First, it used public datasets without verifying or validating the results using *in vitro* or *in vivo* models. Thus, the glycosylation-related signature must be validated in large-scale prospective studies to demonstrate its robustness. Second, our nomogram was not externally validated because of the lack of specific clinical data for ICGC. Furthermore, our results were inconsistent with respect to immune infiltration, tumor stemness, and ICB. Nevertheless, there is no gold standard approach in the growing field of GRGs and their role in cancer. Therefore, we integrated as many analytic strategies and sources as possible to solidify our findings.

In conclusion, our findings indicate that pancreatic cancer and normal tissues express differential GRGs and that pancreatic cancer samples can be divided into four subgroups. We established a prognostic model based on SDC1/TUBAC/SEL1L with favorable prediction performance for pancreatic cancer. The prognostic signature significantly correlated with clinical outcomes, immune infiltration, and pathway enrichment. The findings of this study provide insights into the role of GRGs in the prediction of clinical outcomes in pancreatic cancer.

## MATERIALS AND METHODS

### Screening and integrating data

TCGA (https://portal.gdc.cancer.gov/), GEO (https://www.ncbi.nlm.nih.gov/geo/), and the ICGC (https://dcc.icgc.org/) databases were used to obtain transcriptome fragments per kilo base per million mapped reads (FPKM) data and clinical details. The Genotype-Tissue Expression (GTEx) dataset of normal pancreatic samples was obtained from the UCSC Xena website (https://xenabrowser.net/) to investigate differentially expressed genes. Additionally, 636 GRGs were downloaded from the Molecular Signatures Database (MSigDB, http://www.gsea-msigdb.org/gsea/msigdb/). We then extracted GRGs that co-existed in TCGA, GSE16515, and GSE15471 datasets for further analysis. Mutations in GRGs in pancreatic cancer were analyzed using cBioPortal (http://www.cbioportal.org/). Prognostic model construction and validation relied on TCGA, GEO, and ICGC cohort datasets using log_2_(x + 1) to ensure that gene expression levels were maintained across training and testing. Moreover, GRG mutations in pancreatic cancer were examined using cBioPortal (https://www.cbioportal.org/).

### Enrichment analysis

R (version 4.0.5)’s limma package was used to compare DE-GRGs in pancreatic cancer tissues to those in healthy pancreatic tissues. The DE-GRG screening thresholds were set to |logFC| > 1 and *p* < 0.05. Cluster profiles were used to enrich genes with GO and KEGG to identify potential signaling pathways involved in DE-GRGs. Spearman’s correlation was applied using the R package ggplot2 to detect correlations between DE-GRGs. *p* < 0.05 were regarded as significant (^*^p < 0.05).

### Consistent clustering of molecular subgroups

A consensus analysis was performed using the Consensus Cluster Plus package of R [59] and a survival analysis to compare the prognostic differences between the subgroups. The PAC structure identifying the default cluster number is repeated 100 times to extract 80% of the sample with clusterAlg = “hc,” and innerLinkage = “ward. D2”. A heatmap (v1.0.12) was used to analyze clustering with R software 4.0.3. Gene expression heat maps were compiled for genes with variance greater than 0.1. The top 25% of genes were extracted and displayed after sequencing according to variance depending on the input target gene number. We compared different subtypes and clinical features using ggplot2 and heatmaps from R (v4.0.3). The threshold for statistical significance was fixed at *p* < 0.05.

### Assessment of immune infiltration, immune checkpoint genes (ICGs), response prediction, and stemness

CIBERSORT (a component of the R software package immunedeconv (https://grst.github.io/immunedeconv)) was used to examine the immune infiltration of various subtypes [22]. The relationship between GRG expression and eight common ICGs was examined (SIGLEC15, TIGIT, CD274, CTLA4, HAVCR2, LAG3, PDCD1, PDCD1LC2, and HAVCR2). Immune checkpoint blockade treatment responses were predicted using the TIDE algorithm (ICB) [22]. The mRNAsi was determined using an OCLR approach [24]. We identified the gene expression profile of 11,774 genes using the mRNA expression signature. Spearman’s correlation analysis was performed on RNA expression data. The minimum value was subtracted from the maximum value, and the result was divided by the maximum value to assign a range of (0,1) to the dryness index. Visualization was accomplished using the R software tools ggplot2 and heatmap (v4.0.3).

### Prognostic signature establishment and validation

We used the survival R package and the log-rank test to conduct a Kaplan–Meier analysis to determine the prognostic value of the DE-GRGs on overall survival. *P* < 0.05 were regarded as significant in statistics. A prognostic model was created using LASSO Cox regression analysis of DE-GRGs from the Ggrisk program [60]. A regression coefficient (β) was developed from multivariate Cox regression analysis, and the Prognosis Index (PI) = (β_mRNA1_ × expression level of mRNA1) + (β_mRNA2_ × expression level of mRNA2) + … + (β_mRNAn_ × expression level of mRNAn). The patients were divided into low- and high-risk subgroups in accordance with their median risk scores. The prediction power of the prognostic model was evaluated using Kaplan–Meier survival analysis. A time-related ROC analysis was carried out to assess the prognostic capacity of the risk model using the timeROC program.

The ICGC database was used to validate the glycosylation-related signature risk-score model. The risk scores were calculated in the same manner, and patients were categorized based on the median risk score. Kaplan–Meier and ROC curve analyses were performed. The clinical benefit of the prognostic signature was assessed using DCA [61].

### Developing and evaluating a predictive nomogram

Univariate and multivariate Cox proportional hazards regression analyses were used to determine whether the predictive power of the prognostic model was independent of conventional clinical characteristics. The 1-, 3-, and 5-year survival probabilities of pancreatic cancer were evaluated using an independent prognostic factor nomogram [62]. Calibration plots were used for internal validation to verify their accuracy.

### Analysis of drug response and sensitivity in GRGs

Glycosylation-related genes were analyzed using GSCALite for drug sensitivity [63]. Correlations were determined by Spearman’s correlation analysis between mRNA expression levels of GRGs and the 50% inhibitory concentration values for small molecules against various cells in the GDSC database.

### Evaluation of GRG expression

The expression of GRGs was evaluated using ggplot2 and the ggdendro package to visualize expression in cells from the CCLE (http://www.broadinstitute.org/ccle) database. HPA (https://www.proteinatlas.org/) provided immunohistochemistry data for clinical samples.

### Quantitative real-time PCR (qRT-PCR)

Validation of the mRNA expression levels of SDC1/SEL1L/TUBA1C in PC cell lines (AsPC-1, BxPC-3, PANC-1) and a control cell line (hTERT-HPNE) was done. The PC cell lines were kindly provided by Suzhou Haixing Biosciences Co., Ltd. (Suzhou, China), and the normal pancreatic cell line was obtained from Wuhan Sunncell Biotech Co., Ltd., (Wuhan, China). The PC cell lines and hTERT-HPNE were maintained in RPMI 1640 media with 10% Gibco FBS. All the cells were cultured at 37°C with 5% CO2. Total RNA were isolated from cells using the SPARKeasy bacterial/cell RNA kit (Sparkjade, Shandong, China) and reverse transcription was subsequently performed using the HiScript III RT SuperMix for qPCR (+gDNA wiper) (#R323, Vazyme, China). qRT-PCR was performed with ChamQ Universal SYBR qPCR Master Mix (#Q711, Vazyme) on a QuantStudio™ 1 Plus System (Thermo Fisher Scientific, USA). GAPDH mRNA was employed as a reference gene for normalization of expression, and the 2^−ΔΔCt^ method was applied to quantify changes in the expression of target mRNAs within samples. Primer sequences are listed in Supplementary Table 4. All experiments were repeated at least three times.

### Statistical analysis

Log-rank, Chi-square, Wilcoxon, and Kruskal-Wallis tests were used to compare the variables. The threshold for statistical significance was fixed at *p* < 0.05. R was used to perform all statistical analyses.

## Supplementary Materials

Supplementary Figures

Supplementary Table 1

Supplementary Table 2

Supplementary Table 3

Supplementary Table 4
