# Supplementary material for: A glycosylation-related signature predicts survival in pancreatic cancer
Source: Aging (Albany NY). 2023 Nov 30;15(23):13710–37. doi: 10.18632/aging.205258 (PMC10756102; doi:10.18632/aging.205258)
Supplement: Supplementary Figures [file aging-15-205258-s001.pdf]

SUPPLEMENTARY FIGURES

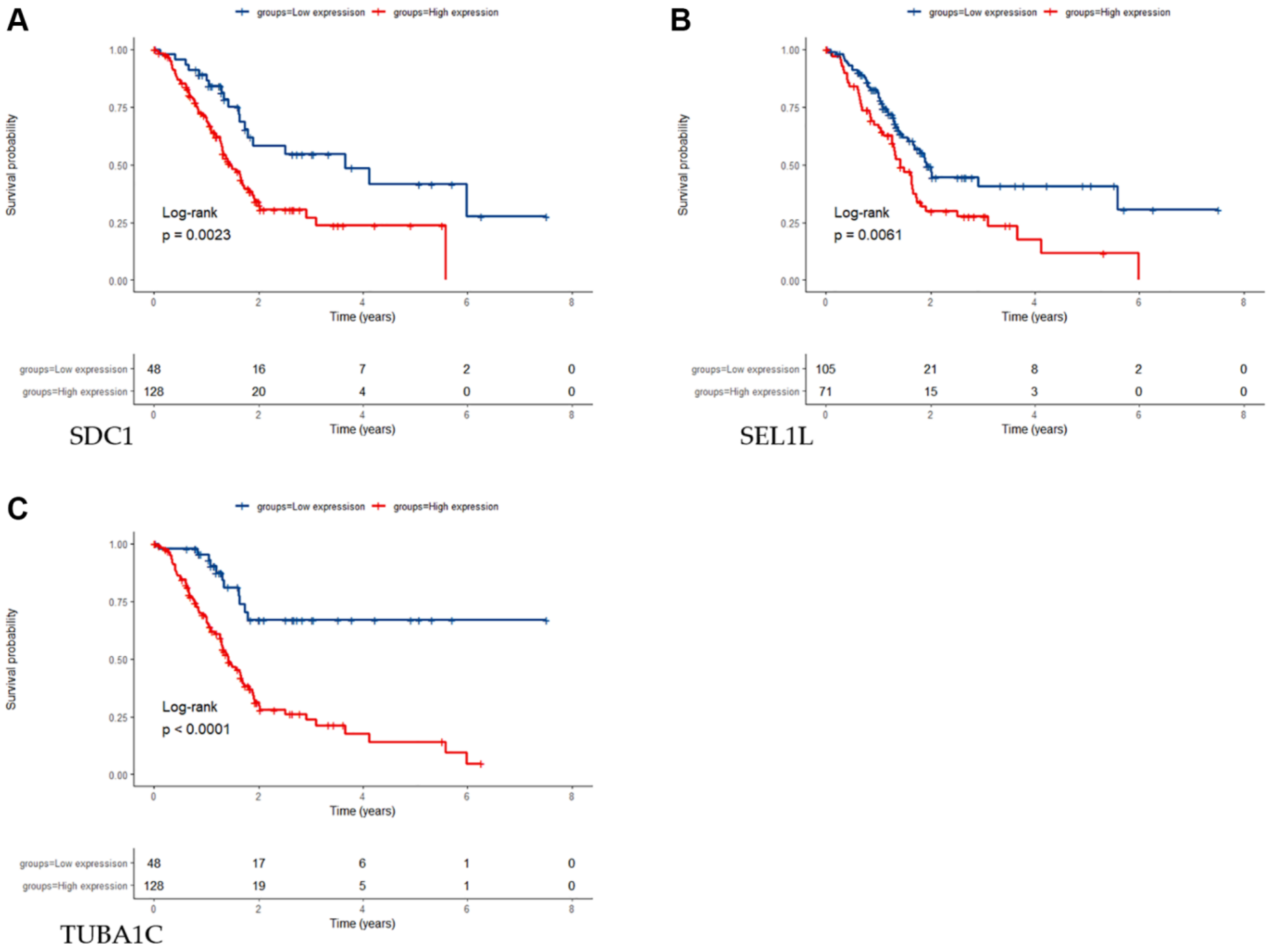

Supplementary Figure 1. Relationship between the expression of three glycosylation-related genes and the prognosis of PC patients. Survival analyses using the TCGA-pancreatic cancer dataset with the expression of SDC1 (**A**), SEL1L (**B**), TUBA1C (**C**).

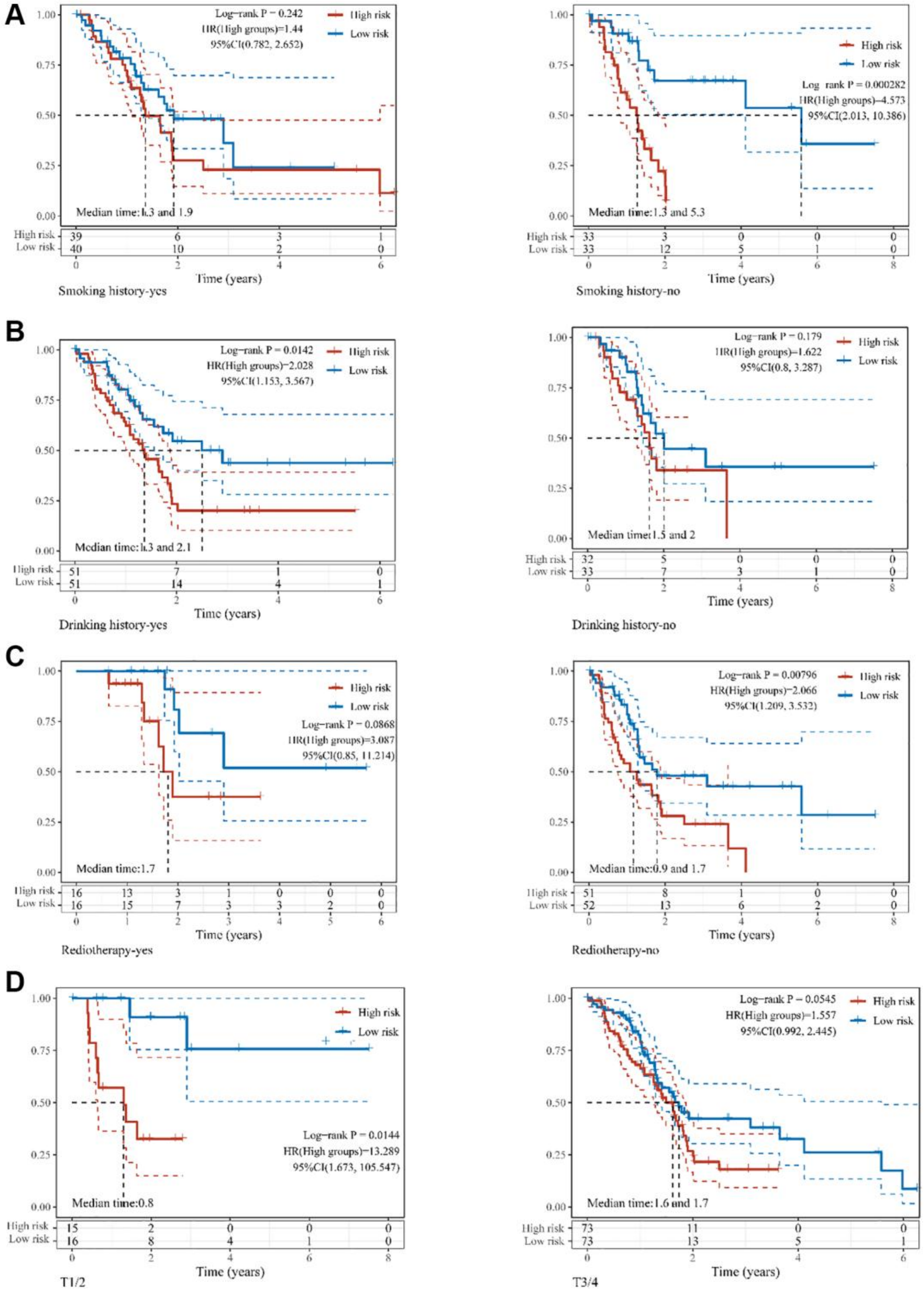

Supplementary Figure 2. Survival curves stratified by different clinical features in TCGA. Survival analyses using the glycosylation-related signature in patients with different smoking history (**A**), drinking history (**B**), radiotherapy history (**C**), and T staging (**D**).

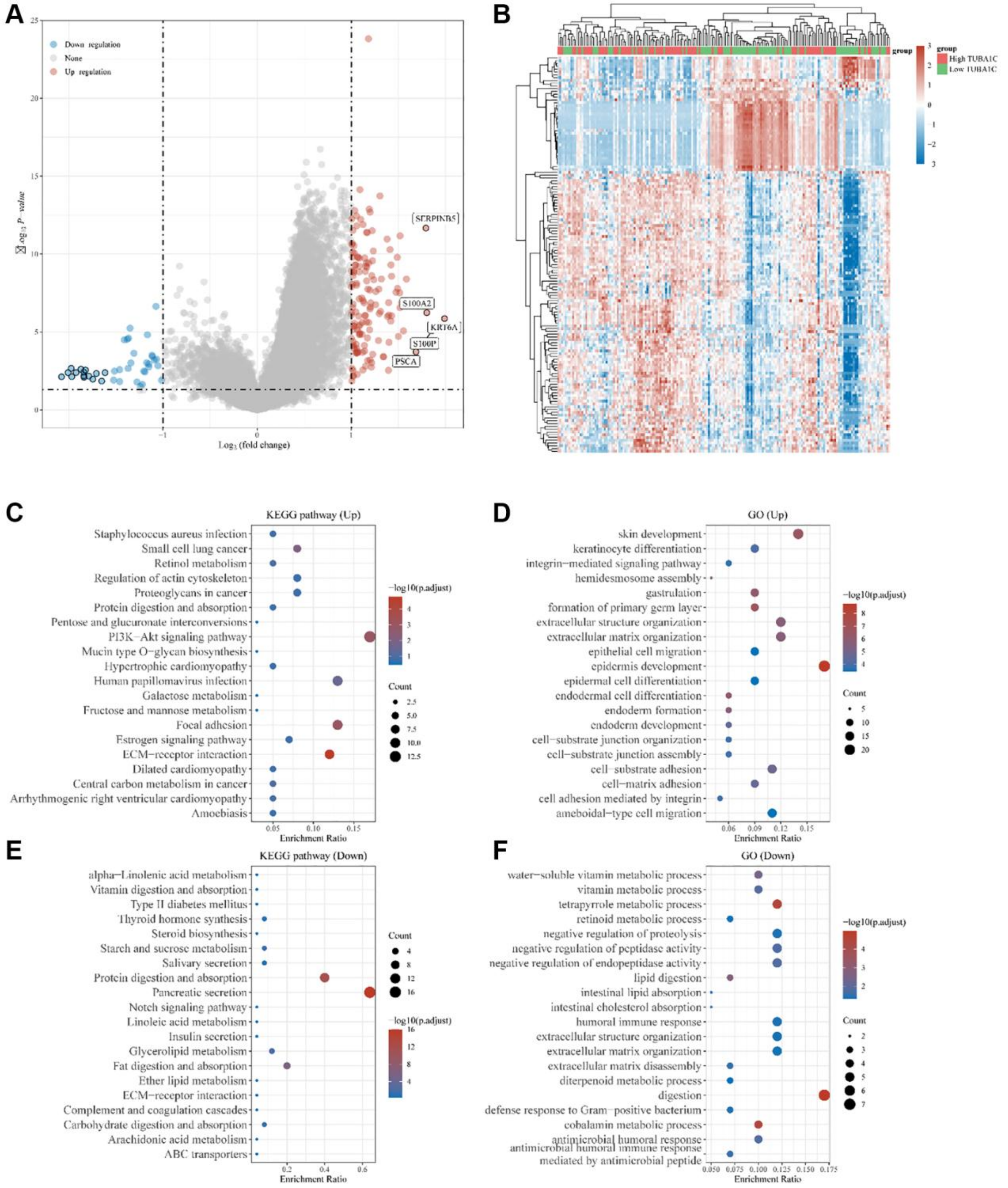

Supplementary Figure 3. Differential expression and enrichment analysis of high and low TUBA1C expression groups. (**A**) Volcano plots show the differential gene expression between TUBA1C high expression and TUBA1C low expression groups using fold-change values and adjusted *p*-values. (**B**) Heatmap showing differential gene expression (only 50 genes were displayed due to a large number of genes). (**C**–**F**) KEGG and GO analyses revealed the signaling pathways associated with up-regulated and down-regulated genes in the TUBA1C high/low expressed groups; an enriched pathway is considered when $p < 0.05$ or FDR <0.05.

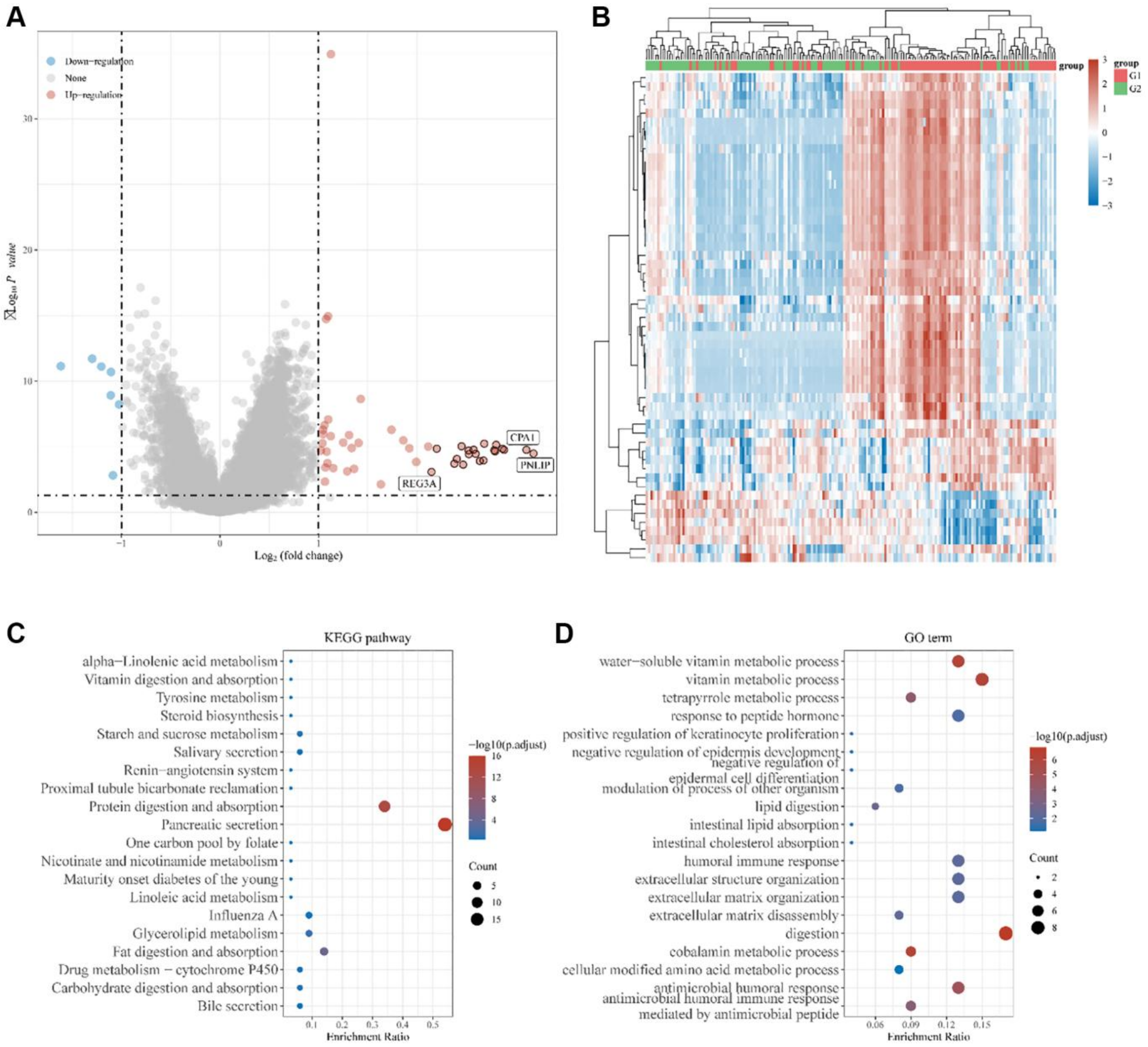

Supplementary Figure 4. Differential expression and enrichment analysis of high and low SEL1L expression groups. (**A**) Volcano plots show the differential gene expression between SEL1L high expression and SEL1L low expression groups using fold-change values and adjusted *p*-values. (**B**) Heatmap showing differential gene expression (only 50 genes were displayed due to a large number of genes). (**C**, **D**) KEGG and GO analyses revealed the signaling pathways associated with up-regulated and down-regulated genes in the SEL1L high/low expressed groups; An enriched pathway is considered when $p < 0.05$ or FDR <0.05.

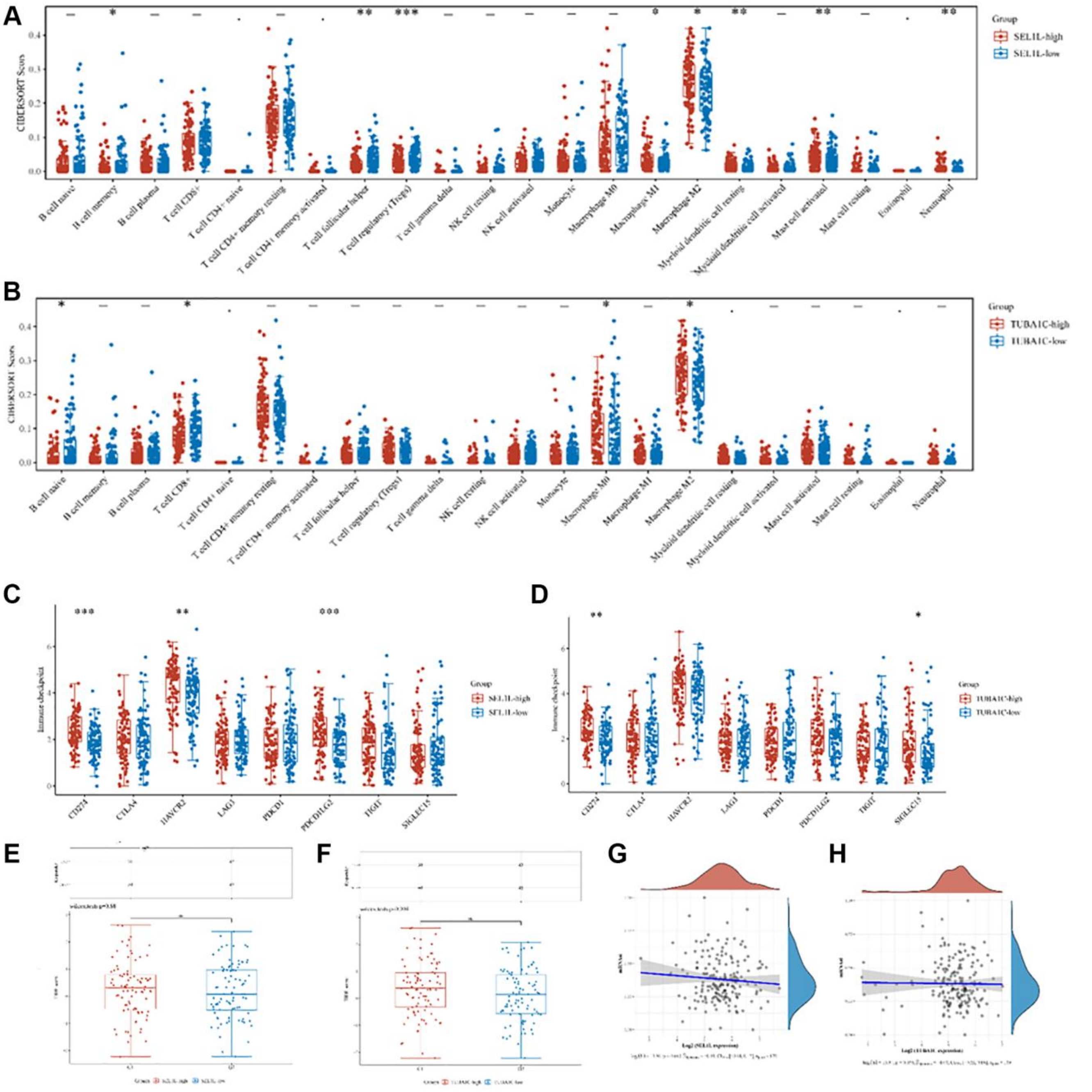

Supplementary Figure 5. Comparison of immune status and stemness between groups that express high and low levels of SEL1L/TUBA1C. (**A**) An analysis of immune infiltration obtained using the CIBERSORT algorithm for the high/low expression group of SEL1L. (**B**) Comparison of immune-checkpoint gene expression in the high/low expression group of SEL1L. (**C**) Table showing immune response statistics and immune response scores for the high/low expression group of SEL1L. (**D**) Stemness for the high/low expression group of SEL1L. (**E**) An analysis of immune infiltration obtained using the CIBERSORT algorithm for the high/low expression group of TUBA1C. (**F**) Comparison of immune-checkpoint gene expression in the high/low expression group of TUBA1C. (**G**) Table showing immune response statistics and immune response scores for the high/low expression group of TUBA1C. (**H**) Stemness for the high/low expression group of TUBA1C. (**A** and **E**) The horizontal axis represents immune cells, while the vertical axis displays immune scores ($^{*}p < 0.05$, $^{**}p < 0.01$, $^{***}p < 0.001$).
