# Supplementary material for: A glycosylation-related signature predicts survival in pancreatic cancer
Source: Aging (Albany NY). 2023 Nov 30;15(23):13710–37. doi: 10.18632/aging.205258 (PMC10756102; doi:10.18632/aging.205258)
Supplement: Supplementary Table 4 [file aging-15-205258-s005.pdf]

SUPPLEMENTARY TABLE

Supplementary Table 4. Primers of genes.

SDC1	F: GAGCTGAAAGGCCGGGAAC<br>R: CTGCTCGATGCTCTCTTGGG
SEL1L	F: GACTCCTTGCACTAACGCGA<br>R: TGCAAAAGGAAATGGTGATGTGT
TUBA1C	F: AATGGAGCAGCTGAGGGAAC<br>R: GCACTCACGCTTGTAAATGGG
GAPDH	F: GGAGTCCACTGGCGTCTTCA<br>R: GTCATGAGTCCTTCCACGATACC
